# Evaluation of Milk Colostrum Derived Lactoferrin of Sahiwal (*Bos indicus*) and Karan Fries (Cross-Bred) Cows for Its Anti-Cancerous Potential

**DOI:** 10.3390/ijms20246318

**Published:** 2019-12-14

**Authors:** Ankita Sharma, Umesh K Shandilya, Monika Sodhi, Ashok K Mohanty, Pranay Jain, Manishi Mukesh

**Affiliations:** 1 ICAR-National Bureau of Animal Genetic Resources, Karnal 132001, India; ankitasharma21@gmail.com (A.S.); ukshandilya@gmail.com (U.K.S.); monikasodhi@yahoo.com (M.S.); 2 University Institute of Engineering and Technology, Kurukshetra University, Kurukshetra 136118, India; drpranayjain@gmail.com; 3 Department of Animal Biosceinces, University of Guelph, Guelph, ON N1G 2W1, Canada; 4 ICAR-National Dairy Research Institute, Karnal, Haryana 132001, India; ashokmohanty1@gmail.com

**Keywords:** anti-cancer, cellular assays, colostrum, gene expression, bovine lactoferrin, native cows

## Abstract

Lactoferrin (Lf) is an iron-binding glycoprotein protein known to have immune-modulatory role and recently, its anticancerous effect against different cancer cell types was emphasized. In the present investigation, a comparative evaluation of anticancer potential of colostrum-derived lactoferrin from Indian native zebu cow (Sahiwal, SAC), crossbred (Karan Fries, KFC) and commercially available (C-Lf) lactoferrin from exotic cow using cellular models was made. A protocol was standardized successfully to purify Lf protein from colostrum of both breeds using HPLC and purity was confirmed by LC–MS. A standardized dose of 750 µg/mL Lf was used to treat two cell types MDA-MB-231 and MCF-7 with Lf from three different sources; SAC-Lf, KFC-Lf and C-Lf for 48 h and 72 h. Different cellular parameters including cytotoxicity, viability, apoptosis and cell proliferation were determined. Comparatively, Lf from commercial source (C-Lf) had maximum effect in both cell types followed by SAC-Lf and KFC-Lf. Further, transcriptional changes in genes associated with apoptosis (*Bax* and *Bcl-2*), tumor progression (*p53*, *p21*, *CD44* and *NF-κβ*) and survival (*survivin*) were evaluated in Lf treatment. The overall results strongly emphasized to the fact that Lf purified from cow colostrum has the capacity to inhibit the in vitro growth of cancerous cell lines albeit to a varied extent.

## 1. Introduction

Cow milk is considered as balanced and nutritive food that becomes an integral part of healthy diet. Cow breeds with difference in genetic makeup have a striking effect on milk composition as well as on the nutritional value of the obtained dairy foods [1,2]. Through series of molecular studies, it has been well demonstrated that *Bos indicus* (zebu) cattle are genetically distinct from their taurine counterparts [3,4,5,6]. India is home to some of the best zebu cattle breeds, which are known for their adaptability to the local agro-climatic conditions, tolerance to harsh climate, tropical diseases and survival under low-input production practices. Indian native cattle breeds are known to have special milk traits producing an important variant of β casein-A2 milk [7,8]. Due to the presence of vital nutrients, cow’s milk has always been a major dietary component and in recent studies, milk from different livestock species have been shown to exhibit a range of biological activities like antimicrobial, antioxidative, antithrombotic, antihypertensive, immuno-modulatory and anti-cancerous [9,10,11,12]. Bovine colostrum, considered to be the most nutritive form of cow milk, is a rich source of bioactive proteins and peptides, immensely involved in the enhancement of immune system with improved defense against various pathogens and diseases [13]. Lactoferrin (Lf) is one of the key proteins in colostrum having an iron-binding ability and known for its antibacterial, antiviral and immune-modulatory properties [14,15,16,17,18,19,20]. The highest concentration of Lf is present in colostrum, albeit varying between species [21]. Lf belongs to the serum transferrin gene family and the bovine Lf gene is localized to chromosome 22 and syntenic group U12 [13,16]. As an iron binding protein, Lf is responsible for its import into cells through receptor-mediated endocytosis. The iron binding ability of Lf in secretions and and circulation, control the free availabity of iron and thus prevents iron precipitation as insoluble hydroxides, inhibit microbial growth and prevent the formation of reactive oxygen species(ROS), responsible for tissue, cell, DNA, protein and membrane lipid damage [22]. To maintain iron homeostasis, complex mechanisms have evolved to regulate cellular and extracellular iron concentrations. Intestinal apical cell membranes are a major site for dietery iron absorption, where it reduced to Fe^2+^ and subsequently transported to enteroctyes eventually exported into the bloodstream via solute carriers. This efflux of Fe^2+^ from enterocytes is regulated by liver hormone-hepicidin, which is known to induce by proinflammatory cytokines and subsequently reduced the iron uptake through systemic iron homoeostasis [22,23]. Therefore, dietery intake and health status of animals play major role in iron metabolism. Lf is a potential gene for milk composition and body measurement traits in dairy cows [20]. Recently, few studies emphasized the protective effect of bovine Lf against head, neck, breast and lung cancer cells [19,21,24]. However, the precise mechanism has not been thoroughly elucidated to date.

As Lf plays a multifunctional role in the immune system, changes at the nucleotide level in the genetic structure could affect its immuno-modulatory activities. In the past, it has been reported that variants of Lf may have a difference in functional properties [25,26,27]. Further, previous studies reported differences among the genetic structure of lactoferrin between *Bos indicus*, cross-bred and the taurine breed [28,29,30,31]. Moreover, the differences in iron-releasing ability are associated with altered functions including anti-microbial, anti-inflammatory and immunomodulatory activities for Lf and iron delivery activity for other transferrins [22]. Therefore, Lf purified from genetically differed cattle breeds might exhibit difference in anti-cancer efficacy. There are various functional single nucleotide polymorphisms (SNPs) identified in bovine Lf among various breeds and using computational approcahes it is speculated that there might be differences in therapeutics potential of protein variants [25,26,27]. In present study, Lf protein purified from colostrum of two main dairy cattle breeds i.e., Sahiwal cattle (*Bos indicus*) ) and Karan Fries cattle (cross between Holstein Friesian cattle (*Bos taurus*) × Tharparkar cattle (*Bos indicus*)) to assess the anticancer potential in breast cancer cell lines. 

## 2. Results

The lactoferrin purified from cow colostrum showed high purity in LC/MS analysis. The Mascot analysis revealed a molecular mass of detected protein to be of 80.12 KDa and the high score of 27666 indicated high purity of Lf. The concentration of Lf purified from colostrum of Sahiwal (SAC) and Karan Fries (KFC) was 6.0 and 1.0 mg/mL, respectively. 

### 2.1. Cytotoxicity Induced by Lf from Different Sources 

**Figure 1:**
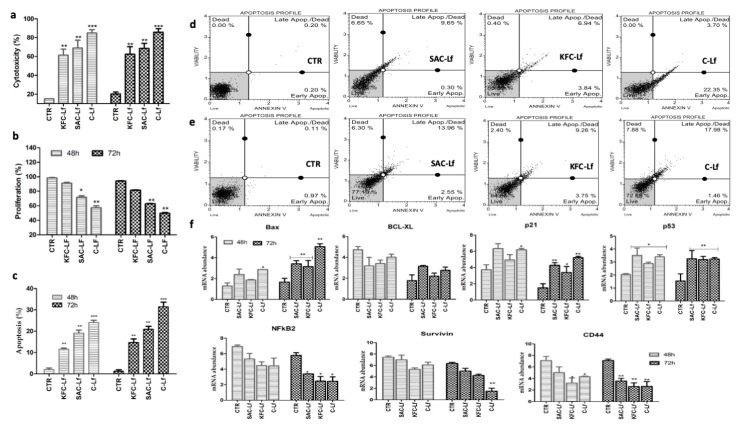
Effect of lactoferrins (Lfs) treatment on MDA-MB-231 cells. (**a**) Induction in cytotoxic levels, (**b**) reduction in cell proliferation, (**c**) increase in apoptosis, (**d**) flow cytometry based annexin V assay graphs at 48 h (**e**) at 72 h and (**f**) relative mRNA abundance of apoptosis, tumor suppressor and cell surface marker associated genes cells following Lf treatment at both a 48 and 72 h time period. Significant difference at *p* < 0.05 is shown by * sign and at *p* < 0.01 by ** and at p < 0.001 by *** sign.

**Figure 2:**
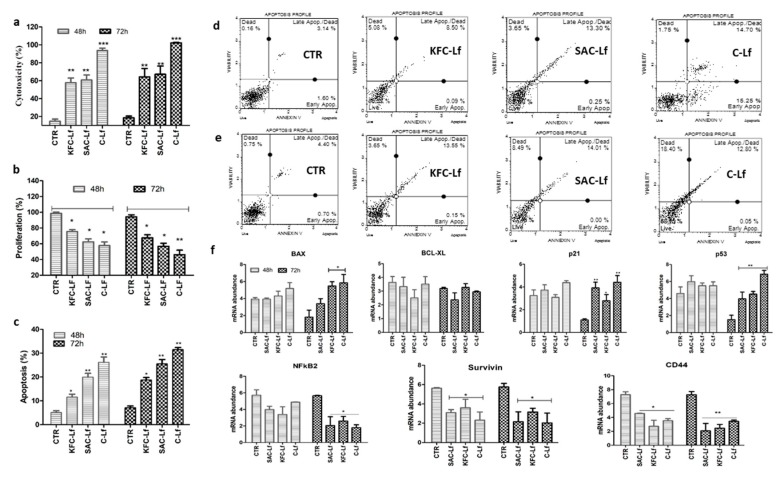
Effect of Lfs treatment on MCF-7 cells. (**a**) Induction in cytotoxic levels, (**b**) reduction in cell proliferation, (**c**) increase in apoptosis, (**d**) flow cytometry based annexin V assay graphs at 48 h (**e**) at 72 h and (**f**) relative mRNA abundance of apoptosis, tumor suppressor and cell surface marker associated genes cells following Lf treatment at both a 48 and 72 h time period. Significant difference at *p* < 0.05 is shown by * sign and at *p* < 0.01 by ** and at *p* < 0.001 by *** sign.

All three sources (SAC-Lf; KFC-Lf and C-Lf (commercial)) of Lf induced significant (*p* < 0.05) cytotoxic level in both cancerous cell types compared to untreated cells (Figure 1a and Figure 2a). C-Lf had a maximum cytotoxic effect on MDA-MD-231 cells with 82% and 90% (*p* < 0.001) (Figure 1a) and on MCF-7 cells with 96% and 105% (*p* < 0.001) cytotoxicity after 48 h and 72 h of incubation, respectively (Figure 2a). SAC-Lf demonstrated 64.9% and 74.4% of cytotoxicity in MDA-MB-231 cells (Figure 1a) and 58.69% and 67.84% of cytotoxicity in MCF-7 cells after 48 h and 72 h, respectively (Figure 2a). While KFC-Lf, showed 61.3% and 65.6% of cytotoxicity in MDA-MB-231 cells and 57.6% and 69.25% of cytotoxicity in MCF-7 cells after 48 h and 72 h, respectively. Therefore, the data showed highest cytotoxicity under C-Lf treatment followed by SAC-Lf and KFC-Lf.

### 2.2. Reduction in Cell Proliferation Treated with Lf from Different Sources

The analysis showed a reduction in cellular proliferation rate in both cell types following treatment with different Lfs compared to control cells. The cell proliferation rate in control (untreated cells) remained higher than 95% in both cell lines during the study. The highest reduction was in the C-Lf treatment followed by SAC-Lf and KFC-Lf (Figure 1b and Figure 2b). In both cell types, the proliferation decreased significantly (*p* < 0.01) in C- Lf treated cells after 48 h and 72 h of incubation compared to the untreated cells. Rate of cell proliferation reduced maximally with the C-Lf treatment to 57.3% and 49.8% on MDA-MD-231 cells (Figure 1b) and to 57.8.54% and 46.25% on MCF-7 cells cytotoxicity after 48 h and 72 h of incubation, respectively (Figure 2b). Similarly, SAC-Lf showed reduction (*p* < 0.05) to 72.7% and 62.5% in treated MDA-MB-231 (Figure 1b) and to 62.4% and 56.8% in MCF-7 cells (Figure 2b). KFC-Lf treated cells showed comparatively less reduction in proliferation rate to 91.2 and 87.3 on MDA-MB-231 cells, but significant reduction (*p* < 0.05) to 75.4% and 67.6% at 48 h and 72 h, respectively was observed on MCF-7 cells (Figure 1b and Figure 2b).

### 2.3. Apoptosis Induced by Lf from Different Sources

A significant induction in apoptosis was detected in both the breast cancer cells treated with Lf derived from different sources in a time-dependent manner (Figure 1c–e and Figure 2c–e). Similar to other cellular parameters, the C-Lf treatment had the most significant effect (*p* < 0.001) on apoptosis in both the cell types, followed by SAC-Lf and KFC-Lf (*p* < 0.01). In MDA-MB-231 cells, the cell death observed for C-Lf, SAC-Lf and KFC-Lf treated groups were 31.44%, 21.16% and, 14.67% respectively, after 72 h of incubation (Figure 1c–e). Similarly, the C-Lf treatment showed maximum cell death in MCF-7 cells with 30.65% followed by SAC-Lf (25.5%) and KFC-Lf (18.77 %) after 72 h of incubation (Figure 2c–e).

### 2.4. Changes at Transcriptional Level

Changes in the expression of genes associated with apoptosis (*Bax* and *BCl2*), tumor progression (*p53, p21, CD44* and *NF-κβ*) and cell survival (*Survivin*) was determined. Similar to other cellular parameters, most of the significant changes in gene expression pattern in both the cancer cells were observed 72 h post-treatment. The induction in *Bax* mRNA expression was significant with a maximum increase in C-Lf (3.4 fold) followed by SAC-Lf (2.05 fold) and KFC-Lf (1.92 fold) treated MDA-MB-231 cells 72 h post-treatment (Figure 1f). Though, in MCF-7 cells, the increase in expression of *Bax* mRNA post-Lf treatment was non-significant, at 48 h post-treatment (Figure 2f), but it increased significantly (*p* > 0.05) only in C-Lf (3.23 fold) and KFC-Lf (3.04 fold) treatment group at 72 h post-treatment. The mRNA abundance of *Survivin* gene decreased in Lf treated MDA-MB-231 and MCF-7 cells. At 72 h, its expression reduced significantly (*p* < 0.05) by 2.5–2.8 fold in MCF-7 cells treated cells in order C-Lf > SAC-Lf > KFC-Lf treated groups (Figure 2f). On the other hand, in MDA-MB-231, significant (*p* < 0.01; 3.87 fold) reduction in expression of *Survivin* gene was observed in only the C-Lf treated group at 72 h post-treatment (Figure 1F). The expression of B-cell lymphoma 2 (*Bcl-2*) an anti-apoptotic gene, showed no statistically significant changes in its expression in Lf treated and untreated groups in both the cell types. On the other hand, mRNA expression of *p53* showed a significant (*p* < 0.05) increase in both cell types at 72 h post-treatment In MDA-MB-231 cells, *p53* expression was significantly (*p* < 0.05) induced (~2 fold) after 72 h in C-Lf, SAC-Lf and KFC-Lf treated groups (Figure 1f). However, in MCF-7 cells, C-Lf treatment group of MCF-7 cell resulted in maximum induction (4.5 fold) of *p53* gene as compared with SAC-Lf (2.7 fold) and KFC-Lf (3.0 fold) at 72 h (Figure 2f). Similarly, the *p21* gene induced significantly (*p* < 0.05) in MDA-MB-231 cells at 72 h with 3.72 fold increased in C-Lf, 2.85 fold in SAC-Lf treated group and 2.42 fold in KFC-Lf treated group (Figure 1f and Figure 2f). Similarly, MCF-7 treated cells also showed a significant (*p* < 0.01) induction in *p21* gene expression with four fold, 3.5 fold and 2.4 fold increase after treating with C-Lf, SAC-Lf and KFC-Lf at 72 h, respectively (Figure. 2f). In contrast, mRNA of *CD44*, an important cell surface adhesion receptor on several cancerous cells showed significant (*p* < 0.05) reduction in its expression in Lf treated cells at 48 h and 72 h post-treatment. Likewise, the mRNA expression of *NF-κβ* that regulates transcription, cytokine production, and cancer initiation, also decreased significantly (*p* < 0.05) in Lf-treated both cell types after 72 h (Figure 1f and Figure 2f).

## 3. Discussion

Importance of colostrum and its constituents have been well highlighted against several diseases [32,33,34,35]. Recently, milk proteins and their peptides have been shown significant inhibition of the invasion and metastasis of human cancer cells [34,35]. However, the differentially originated proteins and their prospective peptides may have altered properties due to the difference in their origin [33]. Differences in the genetic configuration of cattle breeds affect milk yield, composition, proteins and further affect their implications on human health [1,8,36]. Further, it has been reported that different genetic variants of Lf have differed functional properties [25]. The sequences of the lactoferrin gene of native cattle and their comparison with taurine breed were reported in earlier studies [28,30,31]. The present investigation contributed in highlighting the potential implication of Lf purified from colostrum of a native (Indian) cow as an anti-cancerous agent. Although the importance of lactoferrin against cancer was perceived earlier, but now it is drawing the larger attention of researchers as reflected in few recent studies [18,21,37]. The purpose of utilizing colostrum from one native cow and cross-bred cow was based on the fact that genetic makeup of indigenous (zebu) cattle are known to be different compared to exotic cattle. Previous studies have confirmed the differences in milk proteins and their composition in cattle types with a distinct genetic background [1,25,26,27,28,29].

In the present study, colostrum of Sahiwal (native breed) and Karan Fries (cross-bred) cattle was used to purify the lactoefrrin. Amongst 43 registerd native cattle breeds of India, Sahiwal cattle included in the study was considered to be the best zebu cattle. Though, its breeding tract is along the Indo-Pak border in Ferozepur and Amritsar districts of Punjab, and Sri Ganganagar district of Rajasthan, India, it is spread widely across the country. Sahiwal is a heavy breed with a symmetrical body, and burnished red colour body. The breed is most suitable for use in tropical dairy areas and has specific attributes like heat tolerance, tick resistance, bloat tolerance, drought resistance, ease of calving and high milk production. The lifetime milk yield ranges from 2555.67 ± 35.02 kg. to 7712.01 ± 370.0 kg with overall weighted mean of 6438.09 kg. Milk quality is good with 3.4% protein level and 4% butterfat. On the other hand, Karan Fries cattle is a major cross-bred of India developed by crossing between Tharparkar (*Bos indicus*) and Holstein Frisean (*Bos taurus*) cattle. This cross-bred was developed by National Dairy Research Institute Karnal, a premier animal science institute under Indian Council of Agricultural Research, Govt. of India. KFC with an average milk production of 3585 kg is quite popular amongst dairy farmers of North India.

The results of the present study is in agreement with other studies that stated increased cytotoxicity in various cancerous cell lines upon Lf treatment [18,19,20]. Comparatively, SAC-Lf had a slightly higher cytotoxic effect on MDA-MD-231 and MCF-7 cells after 48 h and 72 h of incubation in comparison to KFC-Lf. The exact reason for difference in inducing cell cytotoxicity level by Lf derived from different sources is not yet clear and would definitely be of much interest in future. The difference in cellular activity of Lfs from different sources could be due to a difference in their iron or metal binding efficiacy or due to conformational changes in its structure [22]. Interestingly, in the present investigation, the commercially available Lf showed highest anti-cancer activity than the two Lfs purified in the laboratory using SAC and KFC colostrum. Recently, few reports have also indicated the difference in anti-cancer efficacy of iron saturated and unsaturated forms of Lf [19,38,39]. Some of the previous studies have suggested that the effect of Lf on cellular parameters might depend upon its extent of iron saturation.

In the recent past, a study showed that Lf induces cell cycle arrest in MDA-MB-231 cells at G2 phase while in MCF cells, it induced cell cycle arrest at the G1 and G2 phases [38]. Similarly, bovine Lf treatment has been shown to induce apoptosis in cells of various types of cancer viz., head and neck cancer, colon and gastric cancer [13,24]. Normally cancer cells tend to avoid cell death by surpassing apoptotic signals. However, Lf treatments make these cancerous cells incapable to avoid apoptotic signals and the apoptotic events get initiated through intrinsic as well as extrinsic pathways [38]. Lf induces extrinsic pathway by activating cell death receptors; *FAS* or *TNFR1*, in turn making a complex with procaspases, initiating the caspase cascade for cell death [38,40]. In the present study, induction of apoptosis in both cell lines after 48 h and 72 h further strengthen the notion that bovine colostrum derived lactoferrin has the potential for anti-cancer activity. Likewise, the expression of *Bax* was induced in Lf treated cells might be due to activation of the intrinsic apoptotic pathway. The treatment with Lf might affect mitochondrial membrane permeabilization as well as activate cytochrome-c, which ultimately leads to induction of *Bax* associated pathway. A similar induction of the *Bax* gene was observed by an iron-saturated and unsaturated form of Lf treated cancerous cell types [18]. In addition, the mRNA level of the *Survivin* gene was decreased in the treated cell, in order C-Lf > SAC-Lf > KFC-Lf. Similar down-regulation of the *Survivin* gene upon treatment with Lf on cancer cells was reported [12]. The cancer cells generally exhibit a high level of *Survivin* gene expression to overcome cell cycle checkpoints [41]. Reduction in its expression in Lf treated cancer cells strongly suggests about the anti-cancer potential of Lfs derived from colostrum of SAC and KFC cows. The decrease in expression of the *Survivin* gene in Lf treated groups further strengthens the fact that it has an important role to play during cancer progression as a member of inhibitor of apoptosis family proteins. IAP. It is known to be an inhibitor of apoptosis by inhibiting both *Bax* and *Fas*-associated cell death pathways [42,43]. Additionally, *Survivin* expression is at a minimal level in normal tissues, therefore, it has become a lead cancer marker for both as a tumor diagnostic, prognostic and as well as for anti-cancer therapies. Contrastingly, the induction of *p53* and *p21* genes could provide an important mechanistic aspect in activating apoptosis or cell death in both cancer cells following Lf treatment. The higher induction of these two genes in MCF-7 cells could be linked to the presence of wild-type *p53* in comparison to MDA-MB-231 cells. Activation of these tumor suppressor genes could lead to cell death/apoptosis by regulating the expression of *Bax* gene. Therefore, induction of the *Bax* gene via *p53* linked activation could be an important regulatory component of bovine Lf in inducing cellular apoptosis. Reduced expression of *CD44* and *NF-κβ* genes in treated cells were in accordance with previous studies and indicated the possibility that these two molecules interact in a special way during breast cancer progression. *NF-κβ* signaling was shown to contribute to cancer progression by controlling transition, metastasis and vascularization of tumors via upregulation of VEGF and its receptors [44,45]. *NF-κβ* strongly affects the proliferation and invasiveness of breast cancer cells by regulating *CD44* expression [45,46]. The repressed expression of *CD44* and *NF-κβ* genes in the present study pointed towards the existence of a similar kind of probabilistic mechanism in Lf treated breast cancer cells.

The overall analysis of the cellular parameters and gene expression changes in both the cancerous cells strongly indicated that Lf purified from bovine colostrum has potential to inhibit the growth of cancerous cells albeit to the varied extent. Further, between the two breast cancer cells, MCF-7 cells were found to be more responsive to Lf treatment as the rate of apoptosis and cell death was relatively higher in comparison to MDA-MB-231 cells. This difference in sensitivity of the two cancerous cells might be due to differences in their genetic architecture. Although commercially available Lf induced maximum changes in various cellular and molecular parameters than the purified ones, still the purified Lfs (SAC-Lf and KFC-Lf) showed potent anti-cancer activity in both breast cancer cell lines. The anti-cancer activity of colostrum purified Lfs might be linked to iron binding and iron chelating properties of lactoferrin. This specific characteristic of Lf causes depletion of iron content in the cellular system, which is essential in the tumor microenvironment to sustain the growth and proliferation of cancerous cells.

## 4. Materials and Methods

### 4.1. Lf Purification

Colostrum samples were collected from six healthy cows (48 months), three each of indigenous Sahiwal cows (SAC) and Karan Fries crossbred cows (KFC; Holstein × Tharparkar) within 24 h of calving. These animals were randomly selected from an experimental herd maintained at cattle yard, National Dairy Research Institute, Karnal, India. The animals were maintained as per the standard practices, relevant guidelines and regulations followed at the institute farm. The cows were fed with a well balanced diet as per Indian Council of Agricultural Resaerch (ICAR) feeding standard for dairy animals (2013). The animals in the farm were routinly vaccinated against foot and mouth disease (FMD) and hemorrhagic septicemia (HS). The cattle yard had all the basic facilities including the loose housing structures adequate for housing over 2500 heads of livestock apart from modern state of art milking parlours (verio tendom and flat barn parlours) and a well equipped veterinary health care facility to cater to the health of the animals.The farm is situated at an altitude of 250 m above mean sea level. Latitude and longitude positions being 29°42″ N and 79°54″ E, respectively. Since lactoferrin is known to present in high concetation during early stages of lactation [47], the colostrum samples were collected to purify the lactoferrin. All the selected cows were healthy, free from milk fever, mastitis or retained placenta and were and in their second parity.

After collection, the colosutum samples from three cows were pooled (1.5 L) and defatted by centrifugation at 4000 g for 20 min at 4 °C and diluted 1:1 with Tris-HCl buffer (50 mM, pH 8). Diluted colostrum was incubated with CM-50 Sephadex beads (7 g/lt) and washed five times with 50 mM Tris-HCl buffer (50 mM, pH 8). The lactoperoxidase was removed with 0.2 M NaCl in 50 mM Tris-HCl buffer (pH 8). The lactoferrin protein was then eluted with 0.5 M NaCl-Tris-HCl (pH 8) using column chromatography. The eluted protein was then further purified through ion-exchange HPLC on prefilled SP-Sepharose beads column using AKTA-Prime plus (GE, Healthcare). The purity of the protein was checked on 12% SDS-PAGE, as described in a previous report [48]. Subsequently, in gel digestion of Lf protein band was performed and destained using 40% ACN and 40 mM NH_4_HCO_3_ at a ratio of 1:1 (*v*/*v*) as per method described earlier (26). Later, these destained protein bands were reduced with 5 mM dithiothreitol (DTT) in 40 mM NH_4_HCO_3_ followed by alkylation with 20 mM iodoacetamide in 40 mM NH_4_HCO_3_. Overnight trypsin digestion (12.5 ng/μL; Promega, USA) was carried out at 37 °C and the digested peptides were extracted, lyophilized and desalted using zip-tip (Millipore, Darmstadt, Germany) following manufacturer’s instruction and stored at −80 °C until MS analysis. The lyophilized peptides were reconstituted in 0.1% formic acid in LCMS grade water and subjected to nano-LC (Nano-Advance, Bruker, Germany) and captive spray-Maxis-HD qTOF (Bruker, Germany) mass spectrometer (MS). The MS scan was carried out at an *m*/*z* range of 400–1400. Peak lists were generated by Otof control (version 24.8) using the Hystar post-processing program. Bio-tools software uses the Mascot database search (2.4.1 Matrix Science, UK) at 1% FDR to characterize proteins by correlating the entries in the International Protein Index (IPI) database (January 2015). The protein concentration was estimated using Bradford protein assay. Commercially available (C-Lf) bovine lactoferrin (Sigma) purified from taurine cattle was used to compare with SAC-Lf and KFC-Lf.

### 4.2. In-Vitro Treatment of Purified Lf on Cancerous Cells

Two human breast cancer lines- MCF-7 and MDA-MB-231 cells procured from National Center for Cell Sciences, Pune, India, were employed in the study. Both cell lines were cultured in Dulbecco’s modified Eagle’s medium (DMEM) supplemented with 10% fetal bovine serum (FBS) and antibiotic- anti-mycotic solutions (Sigma) with 5% CO_2_ at 37 °C. The purified Lf was dialyzed in 1× PBS overnight at 4 °C and filtered from 0.22 µm (Millipore). Both cancerous cell types (MCF-7 and MDA-MB-231) were grown in 96 well plates in triplicates one day prior to the treatment schedule at a concentration of 1 × 10^5^ cells per well. A dose of 750 µg/mL was standardized in a trial using range of doses from 125–2000 µg/mL (unpublished data). Cells were treated at standardized dose (750 µg/mL) from all three different sources of lactoferrin (SAC-Lf, KFC-Lf and C-Lf) for a period of 48 h and 72 h.

### 4.3. Cytotoxicity Assay

The cell cytotoxicity induced by Lf was estimated by quantification of lactate dehydrogenase (LDH) activity in the culture medium using LDH cytotoxicity assay kit (Cayman, MI # 10008882). Briefly, 100 µL of supernatant (media from treated and control cultured cells) was incubated with 100 µL of a solution containing assay buffer, NAD ^+^, lactic acid, INT and reconstituted diaphorase at room temperature. Finally, the LDH activity was measured at 490 nm using a microplate reader (Infinite M200PRO, Tecan Life Sciences).

### 4.4. Cell Proliferation Assay

Changes in the cell proliferation rate post Lf treatment was determined using the CyQUANT assay (Invitrogen, Life Technologies). Media was removed from treated and untreated (control) cells and CYQUANT dye reagent prepared as per manual instructions were incubated for 30 min in dark. The fluorescence was measured at 480 nm excitation and 520 nm emission using microplate reader (InfiniteM200PRO, Tecan Life Sciences).

### 4.5. Cell Viability and Apoptosis Assay

Cell apoptosis was measured using The Muse™ Annexin V and Dead Cell Assay kit (Cat No MCH 100105; Millipore). Both treated and control cells were trypsinized and the cell pellet was dissolved in 1× PBS. One hundred µL of cell suspension and 100 µL of reagent mix (Annexin V and 7-AAD) was incubated for 30 min in dark at room temperature and were analyzed using Muse ^®^ Cell Analyzer (Millipore, Bedford, MA, USA).

### 4.6. Gene Expression Analysis

Total RNA was extracted from Lf treated and untreated (control) cells from both cell types harvested 48 and 72 h post-treatment using a Trizol reagent (Invitrogen, ThermoFischer Scientific) and purified RNA through the RNeasy Mini Kit (Qiagen). cDNA was synthesized using 200 ng RNA by Revertaid First Strand cDNA synthesis kit (Fermentas). qPCR was performed in Step OnePlus equipment (Applied Biosystem) using specific primers of target genes taken from literature (Table 1). All reactions were performed in a reaction volume of 10 µL consisting of 4 µL of diluted cDNA, 0.4 µL each of forward and reverse primer (10 pmol), 2 µL of 5× Evagreen (SolisBiodyne) and 3.2 µL of nuclease-free water. For each gene, samples were analyzed in duplicate (technical replicates) along with a 6-point relative standard curve and the nontemplate control at amplification conditions as follows: 10 min at 95 °C, 40 cycles of 15 s at 95 °C (denaturation) and 1 min at 60 °C (annealing + extension). A dissociation protocol with incremental temperatures of 95 °C for 15 s plus 65 °C for 15 s was used to assess the specificity of qPCR reaction and presence of primer dimers. The data was normalized using *ACTB* and *RS18* genes already identified as suitable reference genes for both cell lines [49]. The mRNA abundance was calculated using the first derivative method [50].

## 5. Conclusions

The overall results strongly emphasized to the fact that Lf purified from cow colostrum had the capacity to inhibit the growth of cancerous cells. Though, the anti-cancer potential of C-Lf, SAC-Lf and KFC-Lf against two breast cancer cells was established; the difference in their anti-cancer efficacy might be attributed to the difference in structural conformation or iron saturation level. Still, the colostrum purified Lfs, especially of SAC (native cows) has shown encouraging results and in future could be further evaluated for its therapeutic potential.

## Figures and Tables

**Table 1:** Primer sequences, their slope, efficiency and regression coefficient of candidate genes analyzed in cells under Lf treatment.

Genes	Primer Sequence	Annealing Temperature	Slope	Efficiency	R^2^
*RS18*	GGATGTAAAGGATGGAAAATACA TCCAGGTCTTCACGGAGCTTGTT	60 °C	−3.191	104.62	0.996
*ACTB*	GGCGGCACCACCATGTACCCT AGGGGCCGGACTCGTCATACT	60 °C	−3.085	94.85	0.989
*p21*	TGGAGACTCTCAGGGTCGAAA CGGCGTTTGGAGTGGTAGA	60 °C	−3.306	100.67	0.998
*p53*	ATCTACTGGGACGGAACAGC GTGAGGCTCCCCTTTCTTG	60 °C	−3.021	111.86	0.992
*Bax*	CCTTTTCTACTTTGCCAGCAAAC GAGGCCGTCCCAACCAC	60 °C	−3.131	105.77	0.981
*BCL-xl*	GATCCCCATGGCAGCAGTAAAGCAAG CCCCATCCCGGAAGAGTTCATTCACT	60 °C	−3.265	102.44	0.988
*Survivin*	AGAACTGGCCCTTCTTGGAGG CTTTTTATGTTCCTCTATGGGGTC	60 °C	−2.984	94.62	0.856
*NFkβ2*	ATGGAGAGTTGCTACAACCCA CTGTTCCACGATCACCAGGTA	60 °C	−3.091	110.75	0.991
*CD44*	TGCCGCTTTGCAGGTGTATT CCGATGCTCAGAGCTTTCTCC	60 °C	−3.28	101.78	0.989

## Abbreviations

Lf	Lactoferrin
SAC	Sahiwal cattle
KFC	Karan Fries cattle
C-Lf	Commercial Lactoferrin
HPLC	High Performance Liquid Chromatography
LC-MS	Liquid Chromatography-Mass Spectrometry
ACTB	ß-actin
RS18	Ribosomal Protein S18
